# Proximal fractures of the humerus in patients older than 75 years of age: should we consider operative treatment?

**DOI:** 10.1007/s10195-013-0273-8

**Published:** 2013-11-15

**Authors:** Marjolein de Kruijf, J. P. A. M. Vroemen, K. de Leur, E. A. M. van der Voort, D. I. Vos, L. Van der Laan

**Affiliations:** Department of Surgery, Amphia Hospital, Molengracht 21, 4818 CK Breda, The Netherlands

**Keywords:** Proximal humerus fracture, Operative treatment, Outcome, Old age

## Abstract

**Background:**

Over 75 % of patients presenting with a proximal humerus fracture are 70 years or older. Very little is known about the outcome after operative treatment of these fractures in very old patients. This study was performed to gain more insight in safety and functional outcome of surgical treatment of proximal humerus fractures in the elderly.

**Materials and methods:**

In this observational study, we analyzed all operatively treated patients, aged 75 or older, with a proximal humerus fracture between January 2003 and December 2008 in our center. Patient selection was on clinical grounds, based on physical, mental, and social criteria. Complications were evaluated. We used the DASH Questionnaire to investigate functional outcome, pain, and ADL limitations.

**Results:**

Sixty-four patients were treated surgically for a displaced proximal fracture of the humerus: 15 two-part, 32 three-part, and 17 four-part fractures. Mean DASH scores were 37.5, 36.9, and 48.6, respectively. Regarding the operative methods, overall good results were obtained with the modern locked plate osteosynthesis (mean DASH 34.4). Prosthetic treatment, mostly used in highly comminuted fractures, often resulted in poor function (mean DASH 72.9). Persistent pain and ADL limitations were more present in more comminuted fractures (64 and 50 % in patients with 4-part fractures vs. 14 % in 2-part fractures). There were no postoperative deaths within 3 months of surgery, and fracture-related and non-fracture-related complication rates were low (non-union 3 %; 1 myocardial infarction).

**Conclusion:**

This study shows that it is safe and justifiable to consider surgical treatment of a severely dislocated proximal humerus fracture in selected patients aged 75 and older.

**Level of evidence:**

According to OCEBM Working Group, Level IV.

## Introduction

Up to 80 % of fractures of the proximal humerus are suitable for conservative management, with the use of a sling and physical therapy [1]. However, if severe fracture dislocation is present, poor functional outcome of conservative treatment can be anticipated and surgical intervention must be considered. With the advent of more advanced implants and improved surgical techniques, surgical interventions for these fractures are gaining more acceptance. Possible advantages include faster mobilization, less pain, and better functional outcome. This is particularly true for younger patients [2].

However, to our knowledge, there is no specific report about the outcome of surgically treated fractures of the proximal humerus in the very old patient.

In the older population, fractures of the proximal humerus are most common after hip and wrist fractures. The incidence in patients over 70 years reaches up to 9 per 1,000 population/year. This represents more than 75 % of all proximal humerus fractures [3, 4].

Especially in view of the ever increasing elderly population, insight into functional outcome in this group is very important. Challenges in treating this population operatively are the presence of osteoporosis, higher risks for surgery, and the difficulties in rehabilitation. Knowledge of the functional outcome in these patients may help in making the best choice of treatment in terms of quality of life and health care costs, giving them a chance to maintain an independent lifestyle.

This study was performed to gain more insight into the outcome regarding postoperative mortality, pain, activities of daily life (ADL) limitations, and functional outcome of surgically treated fractures of the proximal humerus in patients aged 75 years and older.

## Materials and methods

This observational study was based on a single-center retrospective research.

All patients operatively treated for fractures of the proximal humerus, from the age of 75 years and older between January 2003 and December 2008 were included in order to have at least 12 months of follow-up [5]. The selection for operative treatment was first based on the configuration of the fracture, of which dislocation of fragments of the fracture is most important. Second, this selection was based on careful clinical judgment of the treating surgeon, considering the physical, mental, and social condition of the patient. We excluded patients from the study who presented 14 days or more after injury, and patients with multiple fractures of the upper extremity. A total of 64 surgically treated patients were included, with an average age of 80.1 years (SD 3.8) at the time of injury. At the time of the research, 13 patients were lost to follow-up: 10 patients had died and 3 cases lacked contact information. Mean follow-up time was 3.7 years (SD 1.4).

Patient characteristics comprised age, gender, affected side, follow-up period, age at injury, risk of surgery as reflected by ASA-classification [7], and operative technique. Fracture classification was performed according to Neer [8]. This classification is primarily based on the number of main fracture fragments.

Various surgical techniques for reconstruction of the fracture were used. The most frequently applied techniques were open reduction and internal fixation (ORIF) using a locking compression plate [the Proximal Humeral Locking Plate (PHLP) or the Philos Plate, both Synthes, Switzerland], ORIF using K-wires and a tension band construction, or hemi-arthroplasty prosthetic repair (either the Global prosthesis, Johnson & Johnson, USA, or the Articula prosthesis, Matthys, Switzerland). The choice for a specific operative technique was based on several factors: first, patient-related factors, such as frailty and more advanced age, were more often a reason to use a less invasive operative technique such as ORIF using K-wires and a tension band construction; second, surgeon-related factors, such as more experience or education in one specific operative technique.; and third, as the study progressed, the use of a locking compression plate became more popular.

Postoperative rehabilitation protocol consisted of protected active and passive circumduction exercises during the first 4 weeks under supervision of a physiotherapist. Progressive active mobilization after 4 weeks was initiated when control X-rays were satisfactory and showed a stable and unchanged osteosynthesis.

Mortality and fracture-related complications such as nerve damage, wound infection, and non-union were recorded. Also, relevant non-fracture-related complications, such as cerebrovascular accidents, sepsis, or thromboembolic disease were researched from the patient files.

We used the DASH Questionnaire (disability of arm, shoulder, and hand) to investigate functional outcome in 51 patients. The scores retrieved from the questionnaires vary from 0 (a perfect function) to 100 (complete loss of function of the injured limb). Patients with severe psychiatric of physical impairment at follow-up could not be investigated by this questionnaire and were excluded from further analysis (*n* = 8). A selection of the questions regarding pain and ADL limitations were used to evaluate pain and ADL limitations as outcome measures.

## Results

In the 5-year period between January 2003 and December 2008, 64 patients aged 75 years or older were treated surgically for a proximal fracture of the humerus. Patient characteristics are listed in Table 1. Ten patients were deceased by the time of this research, with a mean of 22 months after diagnosis (SD 18 months).

**Table 1:** Patient characteristics

	All	% or (SD)	Functional follow-up	% or (SD)
Patients	64	100	51	67
Age, mean	80.1	(3.8)	81.8	(4.3)
Gender: female	58	91	48	94
Affected side: right	26	41	15	38
Psychiatric/physical impairment	8	12	8	16
ASA score, mean	2.4	(0.8)	2.3	(0.8)
Deceased	10	6.4	–	–
Days after surgery	670	(547)	–	–

This population consisted of 15 patients with a two-part, 32 patients with a three-part, and 17 patients with a four-part displaced fracture of the proximal humerus.

ORIF using LCP osteosynthesis was used in 24 patients (7 two-part, 16 three-part, 1 four-part). ORIF with a tension band construction technique in 15 patients (2 two-part, 10 three-part, 4 four-part). In this group, 8 patients required re-operation due to secondary dislocation. A hemi-arthroplasty was performed in 16 patients (4 three-part, 12 four-part). Nine patients were treated with the use of other surgical techniques for internal fixation: intramedullary nail (*n* = 4), single lag screws (*n* = 3), or conventional (non-LCP) plate osteosynthesis (*n* = 2). The exact distribution of the patients among the groups is set out in Table 3.

In this very old patient cohort, selected on clinical criteria of vitality, there were no postoperative deaths within 3 months of surgery. One patient developed a myocardial infarction postoperatively. Three patients were treated successfully for a urinary tract infection. Wound infection occurred in 1 patient and axillary nerve damage in 1 other patient. Osteonecrosis of the humeral head was not observed in this population. A non-union was observed in 2 patients treated with ORIF using LCP osteosynthesis (3 %). Eight out of the 15 patients initially treated with ORIF using K-wire and a tension band wire required re-operation due to secondary dislocation. In these re-operated patients, neither mortality nor additional morbidity was observed.

In the evaluation of functional outcome in these patients, 43 patients were eligible for analysis. The mean DASH score in patients with a two-part fracture was 37.5 (*n* = 7) and in patients with a three-part fracture, 36.9 (*n* = 22). Patients having suffered a four-part fracture did worse with a mean DASH score of 48.6 (*n* = 14). Also, the occurrence of moderate to severe pain and limitations in ADL increased with increasing number of fracture parts (Table 2).

**Table 2:** DASH, pain, and ADL limitation by fracture classification and surgical technique

	Number of patients	Mean DASH (SD)	Moderate/severe pain (%)	Moderate/severe ADL limitations (%)
Fracture classification				
2-part	7	37.5 (25)	1 (14)	1 (14)
3-part	22	36.9 (22)	7 (32)	9 (41)
4-part	14	48.6 (23)	9 (64)	7 (50)
Surgical technique				
LCP	17	34.4 (19)	2 (12)	3 (18)
K-wire/tension band	9	33.9 (21)	5 (56)	5 (56)
Hemi-arthroplasty	14	72.9 (17)	8 (57)	7 (50)

Analysis of the results according to the operative methods showed an interesting trend (Tables 2, 3). Mean DASH scores in ORIF using either a LCP device (*n* = 17) or a tension band wire technique (*n* = 9), in spite of necessary re-operations, were 34.4 and 33.9, respectively. Hemiarthroplasty patients fared less well, their mean DASH score being considerably higher at 72.9 (*n* = 14). Moderate to severe pain and ADL limitations were less present (12 and 18 %, respectively) in patients treated with LCP.

**Table 3:** Detailed report of the study cohort

Neer	*n* included	*n* follow-up	Repair	*n* included	*n* follow-up	Mean DASH	Moderate/severe pain	ADL limitations
2-part	15	7	LCP	7	3	31.4	0	0
			K-wire/tension band	2 (1× reop)	1 (1× reop)	36.7	0	0
			Hemi-arthroplasty	0	–	–	–	–
			Nail	3	2	65.4	1	1
			Lag screws	2	1	13.3	0	0
			Non LCP	1	0	–	–	–
3-part	32	22	LCP	16	13	36.4	2	3
			K-wire/tension band	10 (7× reop)	6 (3× reop)	39.7	3	4
			Hemi-arthroplasty	4	2	22.9	1	1
			Nail	1	0	–	–	–
			Lag screws	1	0	–	–	–
			Non LCP	1	1	56.6	1	1
4-part	17	14	LCP	1	1	0	0	0
			K-wire/tension band	4 (0× reop)	3	20	2	1
			Hemi-arthroplasty	12	10	60.1	7	6
			Nail	0	–	–	–	–
			Lag screws	0	–	–	–	–
			Non LCP	0	–	–	–	–

## Discussion

Data in the current literature suggest that operative treatment of displaced proximal humerus fractures may have a better outcome compared to conservative treatment, but convincing evidence is not available. Especially in the older patients, considerable pain and functional limitations remain after both treatment modalities [1, 2, 9–11].

The aim of this research was twofold. First, it was performed to study whether our policy of operative fracture management of the proximal humerus fracture in the very old, after deliberate and careful selection, was safe in terms of mortality and morbidity. Second, it was undertaken in order to study whether the functional results justified operative treatment of proximal humerus fractures in older patients. Of course, the first aim was of primordial importance; even if the results after surgery were excellent and far better than conservative treatment, an elevated risk of mortality would raise considerable questions about a justified indication.

All patients in this study were operated only after careful selection on clinical grounds. All somatic risk factors were taken into account in our clinical judgment. Many patients included in this study had cardiovascular or pulmonary comorbidities, as might be expected in this old population. However, patients considered not fit for surgery were treated conservatively with use of a sling. We would like to emphasize that mental vitality and social independency of the patients were also important factors in the decision making to perform surgery in case of a shoulder fracture. In this study, no patient died within 3 months of the operation or re-operation. This suggests that it is safe to perform this surgery in well-selected patients.

In a previous report by Owsley [6], locked plate fixation of humeral fractures in patients older than 60 years was associated with a high rate of screw cutout and revision surgery. Surprisingly, the number of fracture- and non-fracture-related complications in our study was very low. Also in this regard, surgery of the shoulder in the selected very aged appeared to be safe.

Furthermore, the functional results of operative repair in general were satisfactory. As is known from the literature, the functional prognosis becomes worse as the number of fracture fragments increases: 4-fragment fractures generally fare much worse than 2- or 3-part fractures, whether treated conservatively or operatively [7]. This also appeared to be true in this cohort of elderly patients: the postoperative results of the 2- and 3-part fractures were satisfactory to good in most patients, with a mean DASH score in these groups of 37.5 and 36.9, respectively (Table 2). Four-part fractures, often treated with a hemi-arthroplasty, generally had a poor postoperative course with a mean DASH score of 53.9.

As to the type of osteosynthetic repair, some interesting findings were noted. Especially in the patients treated by ORIF with locked compression plates, adequate pain reduction and functional recovery were achieved. Although the tension band group seemed to do quite well, it should be kept in mind that these results were obtained after half of these patients were re-operated. An extra operation in this group of patients is, of course, not desirable considering the peri-operative risks. The tension band technique is often used for the treatment of unstable fractures by less experienced surgeons or in patients who were older or had more comorbidities, since it is less invasive, which resulted in re-dislocation of the fracture fragments in some cases. Often, the re-operations were performed by more experienced surgeons, resulting in an eventually good result. Fortunately, these re-operations did not cause further mortality or morbidity, and ended with good results.

In the studied population, pain experience, ADL limitations, and the overall functional outcome, as measured by the DASH score, is significantly worse after hemiarthroplasty compared to internal fixation. This finding is consistent with the results of prosthetic repair in younger groups [2]. Hemi-arthroplasty was predominantly used in severely displaced and/or highly communitive fractures of the proximal humerus. Our rationale for operation was that highly communitive and dislocated fractures of the proximal humerus, especially in cases of a luxated and avascular humeral head, warranted a prosthetic repair. The use of prosthetic repair was primarily intended for minimizing pain rather than regaining function. However, our study showed that severe pain often persists after hemiarthroplasty. It would be interesting to know how these patients would have fared with conservative treatment.

This study shows that it is safe and justifiable to perform osteosynthetic repair of a severely dislocated proximal humerus fracture in selected patients of 75 years and older. This might diminish pain and ADL limitations and potentially contribute to the preservation of an independent lifestyle.

Further research is needed to elucidate the true impact on the quality of life of the very old patient, and to identify specific groups of patients or fracture types that are less suitable for surgical treatment.
